# A default Bayesian hypothesis test for correlations and partial correlations

**DOI:** 10.3758/s13423-012-0295-x

**Published:** 2012-07-14

**Authors:** Ruud Wetzels, Eric-Jan Wagenmakers

**Affiliations:** Department of Psychology, University of Amsterdam, Weesperplein 4, 1018 XA Amsterdam, The Netherlands

**Keywords:** Bayesian inference, Correlation, Statistical evidence

## Abstract

We propose a default Bayesian hypothesis test for the presence of a correlation or a partial correlation. The test is a direct application of Bayesian techniques for variable selection in regression models. The test is easy to apply and yields practical advantages that the standard frequentist tests lack; in particular, the Bayesian test can quantify evidence in favor of the null hypothesis and allows researchers to monitor the test results as the data come in. We illustrate the use of the Bayesian correlation test with three examples from the psychological literature. Computer code and example data are provided in the journal archives.

## Introduction

A correlation coefficient indicates how strongly two variables are related. The concept is basic, and it comes as no surprise that the correlation coefficient ranks among the most popular statistical tools in any subfield of psychological science. The first correlation coefficient was developed by Francis Galton in 1888 (Stigler, 1989); further work by Francis Edgeworth and Karl Pearson resulted in the correlation measure that is used most frequently today, the *Pearson product–moment correlation coefficient*, or *r* (Pearson, 1920). The coefficient *r* is a measure of the linear relation between two variables, where *r* = −1 indicates a perfectly negative linear relation, *r* = 1 indicates a perfectly positive relation, and *r* = 0 indicates the absence of any linear relation.

In this article, we focus on the two-sided hypothesis test for the Pearson correlation coefficient. The standard (i.e., classical, orthodox, or frequentist) test produces a *p* value for drawing conclusions; the common rule is that when *p* < .05, one can reject the null hypothesis that no relation is present. Unfortunately, frequentist *p* value tests have a number of drawbacks (e.g., Edwards, Lindman, & Savage, 1963;Wagenmakers, 2007). For instance, *p* values do not allow researchers to quantify evidence in favor of the null hypothesis (Rouder, Speckman, Sun, Morey, & Iverson, 2009; Wetzels et al., 2011). In addition, *p* values depend on the sampling plan, and hence, its users may not stop data collection when an interim result is compelling, nor may they continue data collection when the fixed sample size result is ambiguous (Edwards et al., 1963). These drawbacks are not merely theoretical but have real consequences for the way in which psychologists carry out their experiments and draw conclusions from their data.

An alternative to frequentist tests is provided by Bayesian inference and, in particular, the so-called Bayes factor (Jeffreys, 1961; Kass & Raftery, 1995). The Bayes factor computes the probability of the observed data under the null hypothesis vis-a-vis the alternative hypothesis. In contrast to the frequentist *p* value, the Bayes factor allows researchers to quantify evidence in favor of the null hypothesis. Moreover, with the Bayes factor, “it is entirely appropriate to collect data until a point has been proven or disproven, or until the data collector runs out of time, money, or patience" ( Edwards et al., , p. 193). Thus, the Bayes factor altogether eliminates the optional stopping phenomenon, where researchers can bias their results by collecting data until *p* < .05 (e.g., Simmons, Nelson, & Simonsohn, 2011). Researchers are allowed to monitor the Bayes factor as the data come in and stop whenever they feel that the evidence is compelling.

In the field of psychology, interest in hypothesis testing using the Bayes factor has greatly increased over the last years. For instance, a method for variable selection in regression models (Liang, Paulo, Molina, Clyde, & Berger, 2008) is used to develop a Bayesian ANOVA (Wetzels, Grasman & Wagenmakers, in press) and a Bayesian *t* test (Rouder et al., 2009; Wetzels, Raaijmakers, Jakab, & Wagenmakers, 2009); Masson has shown how statistical output from SPSS can be translated to Bayes factors using the BIC approximation (Masson, 2011); Hoijtink, Klugkist, and colleagues have promoted Bayes factors for order-restricted inference (e.g., Hoijtink, Klugkis, & Boelen, 2008).

Perhaps the greatest impediment to the large-scale adoption of the Bayes factor is the lack of easy-to-use tests for statistical models that psychologists use in practice. For example, the test for the presence of a correlation (and partial correlation) is one of the most popular workhorses in experimental psychology, yet many psychologists will struggle to find a Bayes factor equivalent. In this article, we remove this hurdle by providing an easy-to-use Bayes factor alternative to the Pearson correlation test.

In this article, we first discuss the standard, frequentist tests for the presence of correlation and partial correlation. Next, we explain Bayesian model selection in general and then focus on a Bayesian test for correlation and partial correlation that is considered default. By *default* (or *objective*, or *uninformative*), we mean that the test is suitable for situations in which the researcher is unable or unwilling to use substantive information about the problem at hand. Key concepts and computations are illustrated with three examples of recent psychological experiments.

## Frequentist test for the presence of correlation

We discuss the frequentist correlation test in the context of a study where participants were involved in an intensive meditation training program (MacLean et al., 2010). The aim of this program was to investigate whether there is an effect of meditation on visual acuity. To assess visual acuity, participants were asked to judge repeatedly whether a vertical line was long or short. Perceptual threshold was defined as the difference in visual angle between the short and the long lines that allowed the participant to classify the lines correctly 75 % of the time. The main result of the experiment was that the intensive meditation program decreased participants’ perceptual threshold.

**Fig. 1:**
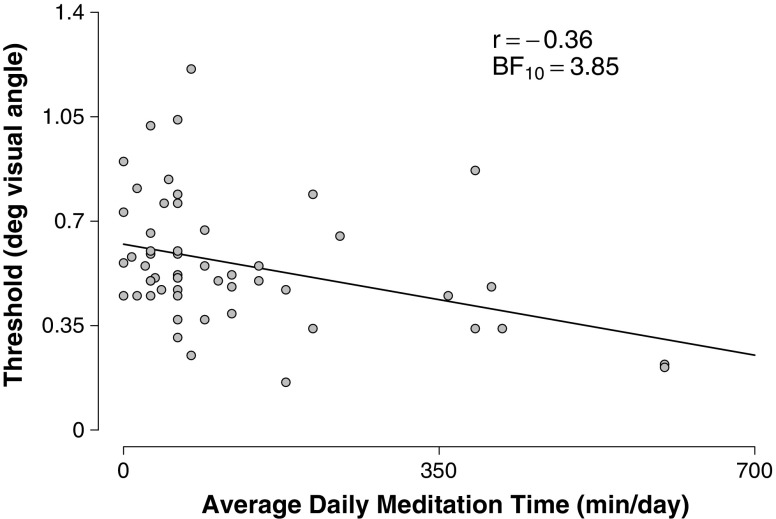
**R**elationship between average daily meditation time and discrimination threshold. A negative correlation suggests that time spent in meditation improves visual perception (i.e., lowers the threshold). Data **are** replotted from MacLean et al, **(** 2010 **)**

In addition to this main result, MacLean et al. (2010) explored whether the improved visual acuity is retained 5 months after termination of the meditation program and, more specifically, whether at follow-up the participants who had meditated the most also had the lowest threshold. The follow-up involved 54 participants, whose data are replotted in Fig. 1. On the basis of these data, MacLean et al. concluded that “this result indicates a correlation between the long-term stability of training-induced discrimination improvement and the maintenance of regular, but less intensive, meditation practice.”

To calculate the correlation between threshold and meditation time, we first define the following variables. For person *i*, mean daily meditation time is denoted *x*_*i*_, and threshold is denoted *y*_*i*_. For meditation time and threshold, the sample variances are $s_X^2 = 20,916.68$ and $s_Y^2 = 0.05$, and the sample means are $\overline x = 121$ and $\overline y = 0.56$, respectively. Then, the sample correlation coefficient of *X* and *Y* is calculated as follows:

1 $$ {r_{XY}} = \frac{{\sum\nolimits_{i = 1}^n \left( {{x_i} - \overline x } \right)\left( {{y_i} - \overline y } \right)}}{{\left( {n - 1} \right){s_X}{s_Y}}} = \frac{{ - 589}}{{1629}} = - .36, $$

where *n* is the number of participants (*n* = 54).

In order to test whether we can reject the null hypothesis that the correlation coefficient is zero, ${\rho_{XY}} = 0$, we calculate the *t* statistic (using ${r_{XY}} = - .36$ and *n* = 54):

2 $$ t = {r_{XY}}\sqrt {{\frac{{\left( {n - 2} \right)}}{{\left( {1 - r_{XY}^2} \right)}}}} = - 2.80, $$

which follows the Student *t* distribution with *n* − 2 degrees of freedom. This *t* statistic corresponds to a *p* value of 0.01. Therefore, with a significance level of *α* = 0.05, researchers may feel that they can confidently reject the null hypothesis of no correlation.

## Frequentist test for the presence of partial correlation

Partial correlation is the correlation between two variables, say *X* and *Y*, after the confounding effect of a third variable *Z* has been removed. Variable *Z* is known as the control variable. In psychological research, there are many situations in which one might want to partial out the effects of a control variable.

Consider a recent experiment on the role of implicit prediction in visual search by Lleras, Porporino, Burack, and Enns (2011). Implicit prediction was studied using an interrupted search task featuring three groups of children and one group of adults (i.e., mean ages of 7, 9, 11, and 19 years). In the search task, participants had to identify a target among a set of distractors (i.e., one “T" among 15 “L" shapes). Crucially, brief looks at the search display (100–500 ms) were interrupted by longer “waits" in which the participant was shown a blank screen (1,000–3,500 ms). The focus of this study was on *rapid resumption*, the phenomenon that, in contrast to the first look at the stimulus (where only 2 % of the correct responses are faster than 500 ms), subsequent looks often show 30 % – 50 % correct responses faster than 500 ms.

On the basis of *n* = 40 observations, Lleras et al., (2011) calculated the correlation between mean successful search time (*X*) and the proportion of rapid resumption responses (*Y*): ${r_{XY}} = .51$, a highly significant correlation (*p* < .01). However, Lleras et al. also observed that this correlation does not take the participants’ age into account. The correlation between search time (*X*) and age (*Z*) is relatively high (i.e., ${r_{XZ}} = - .78$), and so is the correlation between rapid resumption (*Y*) and age (i.e., ${r_{YZ}} = - .66$). Hence, the authors computed a partial correlation to exclude the possibility that age *Z* caused the correlation between search time *X* and rapid resumption *Y*. This is accomplished by the following formula:

3 $$ {r_{XY|Z}} = \frac{{{r_{XY}} - {r_{XZ}}{r_{YZ}}}}{{{{\left[ {\left( {1 - r_{XZ}^2} \right)\left( {1 - r_{YZ}^2} \right)} \right]}^{1/2}}}} = \frac{{.51 - \left( { - .78} \right)\left( { - .66} \right)}}{{{{\left[ {\left( {1 - {{\left( { - .78} \right)}^2}} \right)\left( {1 - {{\left( { - .66} \right)}^2}} \right)} \right]}^{1/2}}}} = - .01. $$

This result shows that by controlling for the variable age, the correlation between search time and rapid resumption is virtually eliminated. The correlation, *r*_*xy*_, is .51, but the *partial* correlation, ${r_{XY|Z}}$, is -.01. The *p* value for the partial correlation can be calculated by computing the *t* statistic (using ${r_{XY|Z}} = - .01$ and *n* = 40):

4 $$ t = {r_{XY|Z}}\sqrt {{\frac{{\left( {n - 3} \right)}}{{\left( {1 - r_{XY|Z}^2} \right)}}}} = - 0.06, $$

which follows the Student *t* distribution with *n − 3* degrees of freedom. This *t* statistic corresponds to a *p* value of .95. Hence, Lleras et al. did not reject the null hypothesis of no correlation between search time and rapid resumption.

Note that this nonsignificant result leaves the null hypothesis in a state of suspended disbelief. It is not statistically correct to conclude from a nonsignificant result that the data support the null hypothesis; after all, the same nonsignificant result could have been due to the fact that the data were relatively noisy. This is one of the prominent *p* value problems that does not occur in the alternative framework of Bayesian inference, which enables researchers to directly gather evidence in favor of the null.

## Bayesian hypothesis testing

In Bayesian model selection or hypothesis testing, the competing statistical hypotheses are assigned prior probabilities. Suppose that we have two competing hypotheses: the null hypothesis, *H*_0_, and the alternative hypothesis, *H*_1_. These hypotheses are assigned prior probabilities of *p*(*H*_0_) and *p*(*H*_1_). Then, after observing the data Y, Bayes’ theorem is applied to obtain the posterior probability of both hypotheses. The posterior probability of the alternative hypothesis, $p\left( {{H_1}|{\text{Y}}} \right)$, is calculated as follows:

5 $$ p\left( {{H_1}|{\text{Y}}} \right) = \frac{{p\left( {{\text{Y}}|{H_1}} \right)p\left( {{H_1}} \right)}}{{p\left( {{\text{Y}}|{H_1}} \right)p\left( {{H_1}} \right) + p\left( {{\text{Y}}|{H_0}} \right)p\left( {{H_0}} \right)}}, $$

where $p\left( {{\text{Y}}|{H_1}} \right)$ denotes the marginal likelihood of the data under the alternative hypothesis (and equivalently for the null hypothesis). The marginal likelihood of the alternative hypothesis is calculated by integrating the likelihood with respect to the prior:

6 $$ p\left( {{\text{Y}}|{H_1}} \right) = \int_\Theta p\left( {{\text{Y}}|\theta, {H_1}} \right)p\left( {\theta |{H_1}} \right)d\theta . $$

Because the posterior model probabilities are sensitive to the prior probabilities of both hypotheses, *p*(*H*_0_) and *p*(*H*_1_), it is common practice to quantify the evidence by the ratio of the marginal likelihoods, also known as the *Bayes factor* (Jeffreys, 1961):

7 $$ \frac{{p\left( {{H_1}|{\text{Y}}} \right)}}{{p\left( {{H_0}|{\text{Y}}} \right)}} = \frac{{p\left( {{\text{Y}}|{H_1}} \right)}}{{p\left( {{\text{Y}}|{H_0}} \right)}} \times \frac{{p\left( {{H_1}} \right)}}{{p\left( {{H_0}} \right)}} = B{F_{10}} \times \frac{{p\left( {{H_1}} \right)}}{{p\left( {{H_0}} \right)}}. $$

The Bayes factor, *BF*_10_, is a weighted average likelihood ratio that indicates the relative plausibility of the data under the two competing hypotheses. Another way to conceptualize the Bayes factor is as the change from prior odds $p\left( {{H_1}} \right)/p\left( {{H_0}} \right)$ to posterior odds $p\left( {{H_1}|{\text{Y}}} \right)/p\left( {{H_0}|{\text{Y}}} \right)$ brought about by the data (cf. Eq. 7). This change is often interpreted as the *weight of evidence* (Good, 1983), and as such, it represents “the standard Bayesian solution to the hypothesis testing and model selection problems" (Lewis & Raftery, 1997, p. 648).

When the Bayes factor has a value greater than 1, this indicates that the data are more likely to have occurred under the alternative hypothesis *H*_1_ than under the null hypothesis *H*_0_, and vice versa when the Bayes factor is below 1. For example, when *BF*_10_ = 4, this indicates that the data are four times as likely to have occurred under the alternative hypothesis *H*_1_ than under the null hypothesis *H*_0_.

Jeffreys (1961) proposed a set of verbal labels to categorize different Bayes factors according to their evidential impact. This set of labels, presented in Table 1, facilitates scientific communication but should be considered only an approximate descriptive articulation of different standards of evidence (Kass & Raftery, 1995).

**Table 1:** Evidence categories for the Bayes factor *BF*_10_ (Jeffreys, 1961). We replaced the label “not worth more than a bare mention" with “anecdotal"

Bayes factor *BF*_10_			Interpretation
	>	100	Decisive evidence for *H*_1_
30	–	100	Very Strong evidence for *H*_1_
10	–	30	Strong evidence for *H*_1_
3	–	10	Substantial evidence for *H*_1_
1	–	3	Anecdotal evidence for *H*_1_
	1		No evidence
1/3	–	1	Anecdotal evidence for *H*_0_
1/10	–	1/3	Substantial evidence for *H*_0_
1/30	–	1/10	Strong evidence for *H*_0_
1/100	–	1/30	Very Strong evidence for *H*_0_
	<	1/100	Decisive evidence for *H*_0_

## Default prior distributions for the linear model

In order to calculate the Bayes factor, one needs to specify prior distributions for the parameters in *H*_0_ and *H*_1_ (cf. Eq. 6). A long line of research in Bayesian statistics has focused on finding appropriate default prior distributions—that is, prior distributions that reflect little information and have desirable characteristics. Much of this statistical development has taken place in the framework of linear regression. In order to capitalize on this work, we later restate the correlation test and the partial correlation test as linear regression:

8 $$ {\text{Y}} = \alpha + \beta {\text{X}} + \varepsilon, $$

where X is the vector of predictor variables, which are assumed to be measured as deviations from their corresponding sample means.

For linear regression, one of the most popular priors is known as Zellner’s *g*-prior (Zellner, 1986). This prior corresponds to a normal distribution on the regression coefficients *β*, Jeffreys’s prior on the error precision *ϕ* (Jeffreys, 1961), and a uniform prior on the intercept *α*:

9 $$ p\left( {\beta |\phi, g,{\text{X}}} \right) = N\left( {0,\frac{g}{\phi }{{\left( {{X^T}X} \right)}^{ - 1}}} \right),p\left( {\phi, \alpha } \right) \propto \frac{1}{\phi }. $$

Note that the information in the data about *β* can be conceptualized as ${\phi^{ - 1}}{\left( {{X^T}X} \right)^{ - 1}}$ (Kass & Wasserman, 1995). Hence, *g* is a scaling factor controlling the information that we give the prior on *β*, relative to the information in the sample. For example, when *g* = 1, the prior carries the same weight as the observed data; when *g* = 10, the prior carries one tenth as much weight as the observed data.

Obviously, the choice of *g* is crucial to the analysis, and much research has gone into choosing an appropriate *g*. This is a difficult problem: A default prior should not be very informative, but a prior that is too vague can lead to unwanted behavior. Various choices of *g* have been proposed; a popular setting is *g* = *n*, the *unit information prior* (*n* equals the sample size; Kass & Wasserman,^1995^), but others have argued for *g* = *k* ^2^ (*k* equals the number of parameters; Foster & George,^1994^) or $g = { \max }\left\{ {n,{k^2}} \right\}$ (Fernandez, Ley, & Steel, 2001). However, the choice for a single *g* remains difficult.

**Fig. 2:**
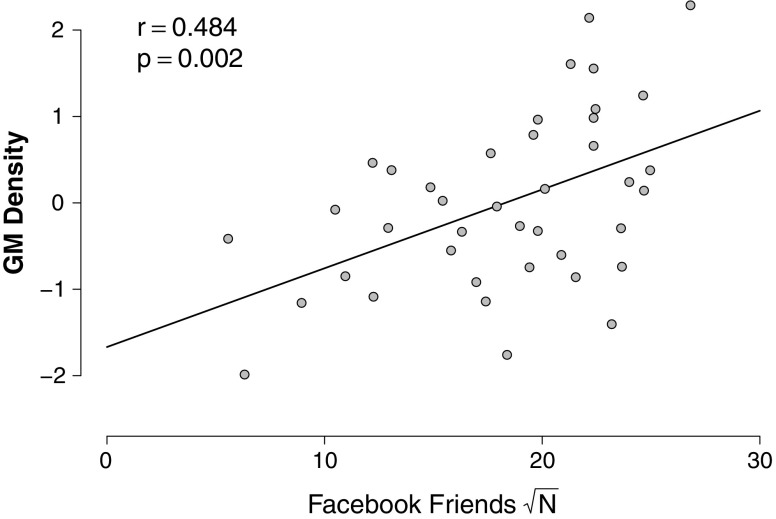
**R**elation between the number of Facebook friends and the normalized gr**a**y matter (GM) density at the peak coordinate of the right entorhinal cortex. A positive correlation indicates that people with many Facebook friends have denser gr**a**y matter in the right entorhinal cortex. Data **are** replotted from Kanai**, Bahrami, Roylance, and Rees (** in press **)**

**Fig. 3:**
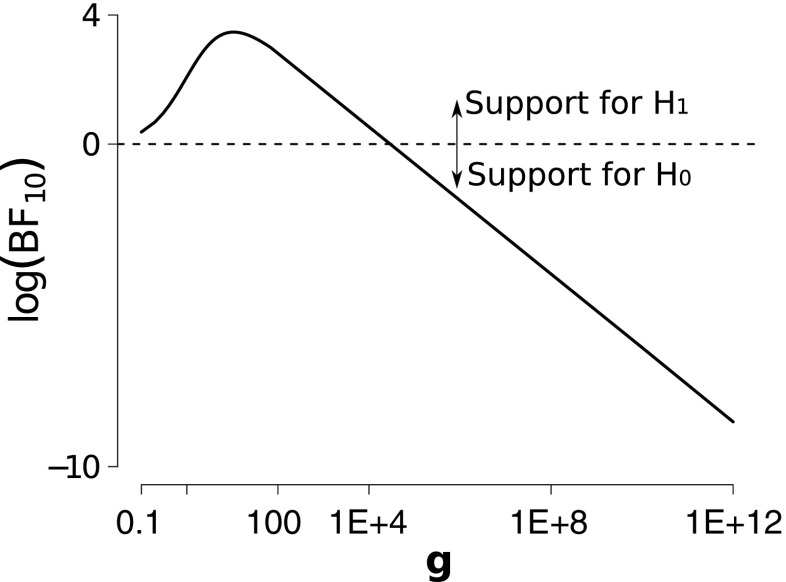
**I**llustration of the Jeffreys**–**Lindley**–**Bartlett paradox when the Zellner *g* prior is applied to the data from Kanai**, Bahrami, Roylance, and Rees** (in press). By increasing *g*, the Bayes factor can be made arbitrarily close to 0, signifying close to infinite support for the null model

The impact of the choice of *g* can be clarified using an example taken from Kanai et al. (in press) that concerned the correlation between the number of Facebook friends and the normalized gray matter density at the peak coordinate of the right entorhinal cortex. Figure 2 shows the data; people with more Facebook friends have higher gray matter density, *r* = .48, *p* < .002. The effect that a specific choice of *g* has on the Bayes factor for this data set is shown in Fig. 3. This figure demonstrates that when *g* is increased, the support for the null hypothesis can be made arbitrarily large. This is due to the fact that if *g* increases, the vagueness of *M*_1_ does too. This phenomenon is known as the Jeffreys–Lindley–Bartlett paradox (Bartlett, 1957; Jeffreys, 1961; Lindley, 1980; but see Vanpaemel, 2010). One of the primary desiderata for a default Bayesian hypothesis test is to avoid this paradox.

In a different but related approach, Zellner and Siow (1980) extended the work of Jeffreys (1961) and proposed assigning the regression coefficients a multivariate Cauchy prior, with a precision based on the concept of unit information (Liang et al., 2008). However, the marginal likelihood for this model specification is not analytically tractable, and therefore, this approach did not gain much popularity (but note that these priors are well-studied nonetheless; see Bayarri & Garcia-Donato, 2007; Berger, Ghosh, & Mukhopadhyay, 2003; Berger & Pericchi, 2001).

Recently, however, Liang et al. (2008) represented this Jeffreys–Zellner–Siow (JZS) prior as a mixture of *g*-priors—that is, an inverse-gamma $\left( {1/2,n/2} \right)$ prior on *g* and Jeffreys’s prior on the precision *ϕ*:

10 $$ \matrix{ {p\left( {\beta |\phi, g,{\text{X}}} \right) = \smallint N\left( {0,\frac{g}{\phi }{{\left( {{{\text{X}}^T}{\text{X}}} \right)}^{ - 1}}} \right)p(g)dg} \hfill \\ {p\left( \phi \right) \propto \frac{1}{\phi }} \hfill \\ {p(g) = \frac{{{{\left( {n/2} \right)}^{1/2}}}}{{\Gamma \left( {1/2} \right)}}{g^{ - 3/2}}{e^{ - n/\left( {2g} \right)}}.} \hfill \\ }<!end array> $$

From this (mathematically equivalent) perspective, the problem of selecting a single *g* has been mitigated by assigning *g* a prior. The formulation above combines the computational advantages of the *g*-prior with the theoretical advantages of the Cauchy prior (see Liang et al., 2008, for details). Moreover, the mixture representation also facilitates the calculation of the Bayes factor, leaving only one integral that has to be estimated numerically. Note that the same prior setup underlies the JZS ANOVA (Wetzels et al., in press) and the JZS *t* test (Rouder et al., 2009; Wetzels et al., 2009). In the following, we will use this setup for our correlation and partial correlation test.

## The JZS Bayes factor for correlation and partial correlation

In order to calculate the Bayes factor for the JZS (partial) correlation test, we conceptualize these Bayesian tests as a comparison between two regression models, such that the test becomes equivalent to a variable selection test for linear regression (i.e., a test of whether or not the regression coefficient *β* should be included in the model). This conceptualization allows us to exploit the JZS prior distribution. Computer code for calculating the JZS Bayes factors is presented in the Appendix.

### The JZS Bayes factor for correlation

Suppose that we have observed data from two variables, *X* and *Y*, and are interested in their correlation. Consider the regression from Eq. 8, where *α* is the intercept, *β* is the regression coefficient, and *ε* is the error term, normally distributed with precision *ϕ*.

Next, we are interested in how well this regression equation fits the data. The standard method for assessing this fit is by calculating the coefficient of determination *R* ^2^:

11 $$ {R^2} = 1 - \frac{{S{S_{err}}}}{{S{S_{tot}}}}, $$

where *SS*_*err*_ denotes the residual sum of squares and *SS*_*tot*_ denotes the total sum of squares. Note that *R* ^2^ is the proportion of variance that is accounted for by the regression model. Specifically, *R* ^2^ is an indication of how much better the fit of model *M*_1_ is, when compared with model *M*_0_:12


When *R* ^2^ is low (i.e., near zero), the addition of the regression coefficient *β* to *M*_0_ has caused only a small increase in explained variance. As *R* ^2^ increases, so does the importance of *β*. Because *R* ^2^ is the square of the sample correlation *r*, a test for whether or not the correlation equals zero is equivalent to a test for whether or not *β* equals zero. Hence, the correlation test can be recast as a comparison between two linear regression models, *M*_0_ and *M*_1_ (e.g., Draper & Smith, 1998).

The Bayes factor *BF*_10_ using the JZS prior setup can then be calculated as follows (see Liang et al., 2008):

13 $$ \matrix{ {B{F_{10}} = \frac{{p\left( {{\text{Y}}|{M_1}} \right)}}{{p\left( {{\text{Y}}|{M_0}} \right)}}} \\ { = \frac{{{{\left( {n/2} \right)}^{1/2}}}}{{\Gamma \left( {1/2} \right)}} \times \int_0^\infty {{\left( {1 + g} \right)}^{\left( {n \;-\; 2} \right)/2}} \times {{\left[ {1 + \left( {1 - {r^2}} \right)g} \right]}^{ - \left( {n \;-\; 1} \right)/2}}{g^{\left( { - 3/2} \right)}}{e^{ - n/\left( {2g} \right)}}dg.} \\ }<!end array> $$

Note that the only input to Eq. 13 is the usual sample correlation *r* and the number of observations *n*. The resulting Bayes factor *BF*_10_ quantifies the evidence in favor of the alternative hypothesis. Therefore, Bayes factors greater than 1 indicate evidence for the presence of a correlation, and Bayes factors smaller than 1 indicate evidence for the absence of a correlation.

### The JZS Bayes factor for partial correlation

Again, we formalize the test as a model selection problem between two regression models. Assume that we have three variables, *Y*, *X*_1_, and *X*_2_, and we want to test whether the partial correlation between *Y* and *X*_2_ is zero or not. Analogously to the correlation example, one is interested in whether adding the variable *X*_2_ increases *R* ^2^ when the variable *X*_1_ is already included in the regression model. Hence, we compare the two models (e.g., Draper & Smith, 1998):14


The Bayes factor *BF*_10_ using the JZS prior setup can then be calculated as follows:

15 $$ \matrix{ {B{F_{10}} = \frac{{p\left( {{\text{Y}}|{M_1}} \right)}}{{p\left( {{\text{Y}}|{M_0}} \right)}}} \\ { = \frac{{\int_0^\infty {{\left( {1 + g} \right)}^{\left( {n - 1 - {p_1}} \right)/2}} \times {{\left[ {1 + \left( {1 - R_1^2} \right)g} \right]}^{ - \left( {n - 1} \right)/2}}{g^{\left( { - 3/2} \right)}}{e^{ - n/\left( {2g} \right)}}dg}}{{\int_0^\infty {{\left( {1 + g} \right)}^{\left( {n - 1 - {p_0}} \right)/2}} \times {{\left[ {1 + \left( {1 - R_0^2} \right)g} \right]}^{ - \left( {n - 1} \right)/2}}{g^{\left( { - 3/2} \right)}}{e^{ - n/\left( {2g} \right)}}dg}}} \\ }<!end array> $$

Input to Eq. 15 is the coefficient of determination for *H*_0_ and for *H*_1_ (i.e., $R_0^2$ and $R_1^2$), the number of regression coefficients *H*_0_ and *H*_1_ (i.e., *p*_0_ and *p*_1_), and the number of observations *n*. Note that the coefficient of determination for *M*_0_ is found by squaring the sample correlation between the variable of interest and the controlling variable: $R_0^2 = r_{Y{X_1}}^2$; the coefficient of determination for *M*_1_ can be written as $R_1^2 = r_{Y{X_2}|{X_1}}^2\left( {1 - r_{Y{X_1}}^2} \right) + r_{Y{X_1}}^2$. As before, the resulting Bayes factor *BF*_10_ quantifies the evidence in favor of the alternative hypothesis.

### Correlation example: The meditation data

In the meditation study, MacLean et al. (2010) tested the hypothesis of a relation between meditation time and visual acuity (see Fig. 1). The sample correlation between these two variables was found to be *r*_*XY*_ = −.36; the associated *p* value is .01, significant at the *α* = .05 level.

We can now apply Eq. 13 to calculate the Bayes factor. Entering *r*_*XY*_ = −.36 and *n* = 54 in Eq. 13 yields a Bayes factor *BF*_10_ = 3.86, indicating that the data are 3.86 times more likely to have occurred under *H*_1_ than under *H*_0_, a “substantial" Bayes factor according to the coarse category scheme proposed by Jeffreys (1961). However, note that the factor 3.86 inspires less confidence than does the *p* value; this illustrates the well-known point that *p* values overestimate the evidence against the null, at least when the *p* value is misinterpreted as the posterior probability of the null hypothesis being true (e.g., Edwards et al., 1963; Wetzels et al., 2011).

### Correlation example: The Facebook data

In the Facebook study, Kanai et al. (in press) investigated the relation between the number of Facebook friends and the normalized gray matter density at the peak coordinate of the right entorhinal cortex (see Fig. 2).

Entering *r*_*XY*_ = .48 and *n* = 40 in Eq. 13 yields a Bayes factor *BF*_10_ = 17.87, indicating that the data are 17.87 times more likely to have occurred under *H*_1_ than under *H*_0_, a “strong" Bayes factor according to the coarse category scheme proposed by Jeffreys (1961).

### Partial correlation example: The rapid resumption data

In the study on rapid resumption, Lleras et al. (2011) tested the partial correlation between search time (*X*) and rapid resumption (*Y*) while controlling for age (*Z*). The partial correlation was found to be *r*_*XY|Z*_ = −.01, with a *p* value of .95.

We can compute the Bayes factor using the coefficient of determination for both models. The null model *M*_0_ regresses search time (*X*) on age (*Z*), containing only the regression coefficient for *Y*. Hence, in Eq. 15, *p*_0_ = 1, and $R_0^2 = 0.6084$. The alternative model *M*_1_ contains the regression coefficients for *Y* and *Z*. Hence, *p*_1_ = 2, and $R_1^2 = 0.6084408$. The sample size *n* is 40.

The Bayes factor *BF*_10_ is 0.13, indicating substantial evidence in favor of the null hypothesis: The data are $1/0.13 \approx 7.70$ times as likely to have occurred under the null hypothesis than under the alternative hypothesis (see Table 1).

## Concluding remarks

In this article, we outlined a default Bayesian test for correlation and partial correlation. Just like the default Bayesian ANOVA (Wetzels et al., in press) and *t* test (Rouder et al., 2009; Wetzels et al., 2009), the correlation test follows directly from the regression framework for variable selection proposed by Liang et al., (2008). We did not strive for new statistical development. Instead, our goal was to show experimental psychologists how they can obtain a default Bayesian hypothesis test for correlation and partial correlation. As we mentioned throughout this article, the Bayesian hypothesis test comes with important practical advantages, as compared with the standard frequentist test; for instance, the Bayesian hypothesis test can quantify evidence in favor of the null hypothesis and allows researchers to collect data until a point has been proven or disproven.

It should be noted that Jeffreys (1961) also proposed a Bayesian correlation test, one that differs slightly from the one outlined here. We prefer the JZS correlation test because it follows directly from the regression framework of Liang et al., (2008), incorporating modern Bayesian developments into a more general JZS testing framework. This JZS framework now encompasses linear regression, the *t* test, ANOVA, and (partial) correlation, and extensions to other popular statistical models are likely to follow.

By making default Bayes factors easily available to experimental psychologists, we hope and expect that the field will start to turn away from *p* values and move toward a Bayesian assessment of evidence. This transition is bound to improve statistical inference and accelerate scientific progress.
